# Acknowledgement of manuscript reviewers 2019

**DOI:** 10.18332/ejm/118137

**Published:** 2020-02-11

**Authors:** Victoria G. Vivilaki

**Affiliations:** 1 Department of Midwifery, University of West Attica, Athens, Greece

## CONTRIBUTING REVIEWERS

The editors of European Journal of Midwifery would like to thank all our reviewers who have contributed to the journal in Volume 3 (2019).

**Alé, Franck**

France

**Αlipour, zahra**

Iran

**Antonakou, Angeliki**

United Kingdom

**Baird, Kathleen**

Australia

**Betron, Myra**

United States

**Bonilla, Hilda**

Chile

**Curtis, Kath**

United States

**Darra, Susanne**

United Kingdom

**Dey, Teesta**

United Kingdom

**Dieb, Amira**

Egypt

**Durain, Dawn**

United States

**Egenberg, Signe**

Norway

**Escuriet, Ramón**

Spain

**Forbes, Faye**

Australia

**Fox, Deborah**

Australia

**Grum, Teklit**

Ethiopia

**Harris, Melissa**

Australia

**Hisano, Michi**

Japan

**Hunter, Billie**

United Kingdom

**King, Rosemary**

Australia

**Lagana, Antonio**

Italy

**Lambert, Jaki**

United Kingdom

**Mackenzie, Ian**

United Kingdom

**Mas, Masresha L.**

Ethiopia

**Mc Carthy, Jane**

Ireland

**Meneses-Rentería, Alba**

Mexico

**Mitchell, Creina**

Australia

**Mivšek, Polona**

Slovenia

**Münstedt, Karsten**

Germany

**Ny, Pernilla**

Sweden

**Oo, Win**

Malaysia

**Prosen, Mirko**

Slovenia

**Sandwell, Rachel**

Canada

**Sharek, Danika**

Ireland

**Teklesilasie, Wondwosen**

Ethiopia

**Tura, Halkeno**

United States

**Vermeulen, Joeri**

Belgium

**Werner, Anette**

Denmark

**Whitburn, Laura**

Australia

**Wier, Jacqueline**

United Kingdom

**Yurdakul, Mine**

Turkey

